# Voltage-Gated Sodium Channel Substitutions Underlying Tetrodotoxin Resistance in Nemerteans: Ecological and Evolutionary Implications

**DOI:** 10.3390/ijms262411785

**Published:** 2025-12-05

**Authors:** Vasiliy G. Kuznetsov, Anna E. Vlasenko, Timur Yu. Magarlamov

**Affiliations:** A.V. Zhirmunsky National Scientific Center of Marine Biology, Far Eastern Branch, Russian Academy of Sciences, 690041 Vladivostok, Russia

**Keywords:** tetrodotoxin, TTX, voltage-gated sodium channels, NaV channels, TTX resistance, Nemertea, ribbon worms, convergent evolution, molecular adaptation

## Abstract

Tetrodotoxin (TTX) is an extremely potent neurotoxin, a selective blocker of voltage-gated sodium (NaV) channels, produced by bacteria and accumulated across a wide range of taxa. Several TTX-bearing animals have developed molecular adaptations in their NaV channels that provide TTX resistance, making this toxin one of the factors of molecular evolution. However, the molecular basis of TTX resistance in NaV channels of a significant proportion of tetrodotoxic species remains poorly studied. Nemertea is a phylum of marine worms, comprising both TTX-bearing and non-TTX-bearing species. Here, we analyzed the amino acid sequences of the NaV1 channel regions responsible for TTX binding from 22 species of nemerteans. Substitutions previously characterized as conferring TTX resistance in other taxa were detected in sixteen nemerteans; local clustering was observed within several families. These findings suggest that TTX resistance in nemerteans evolved multiple times independently and may serve as either as an adaptation facilitating TTX accumulation for subsequent use for defense and predation, or as a mechanism allowing consumption of tetrodotoxic prey without toxin accumulation.

## 1. Introduction

Tetrodotoxin (TTX) is an extremely potent, low-molecular-weight neurotoxin that, along with its analogs (TTXs), is widely distributed among diverse marine and terrestrial taxa, serving both as a predatory weapon and as a defense agent [1,2]. Although the origin of the toxin remains debated, several studies provide evidence supporting its bacterial source [3,4,5]. The variety of tetrodotoxic animals is impressive—from flatworms and gastropods to fishes and newts [6,7].

TTX acts as a selective blocker of voltage-gated sodium (NaV) channels, which are responsible for propagating action potentials [8,9]. NaV channels are large proteins of approximately 2000 amino acids, composed of four homologous domains (DI-DIV) [10]. Each domain contains six transmembrane segments (S1–S6), connected by loops. Segments S1–S4 of each domain function as the voltage-sensing module, while S5 and S6 form the ion-conducting pore. The loops between S5 and S6, known as pore-loops (P-loops), form the selectivity filter and outer vestibule of the channel. TTX binds to the P-loop region of NaV channels, blocking the influx of Na^+^ ions into the cell. Amino acid (AA) substitutions in the P-loops can affect TTX-binding affinity [11,12,13].

In TTX-bearing species, protection against self-intoxication is presumably achieved through structural modifications of NaV channels, primarily via AA substitutions in their P-loop regions [14]. Both TTX accumulation and the adaptive modifications of NaV channels are thought to have arisen independently across multiple taxa, representing a clear case of convergent evolution [11,12,13,14]. Although AA substitutions in P-loop regions vary widely, the total number of possible variants is thought to be limited by the requirement to maintain normal channel functionality [12,15,16].

Most investigations of TTX resistance in NaV channels have focused on highly toxic taxa, mostly vertebrates, while TTX-bearing invertebrates remain neglected. Nemerteans, or ribbon worms, represent a phylum of mostly marine worms comprising three classes: Palaeonemertea, Hoplonemertea, and Pilidiophora [17,18]. A number of nemertean species from all three classes were found to contain TTXs, even in extremely high concentrations [19,20,21,22]. Recently, it was demonstrated that the low-toxic heteronemertean *Kulikovia alborostrata* possesses two AA substitutions in the P-loop regions of the NaV1 channel, presumably decreasing TTX binding affinity [23]. However, no other nemertean NaV channels have been investigated to date.

Here, we identify AA substitutions in NaV channels that are potentially associated with TTX resistance and analyze their distribution across the nemertean phylum. We also discuss the potential relationship between TTX levels and structural variations in the P-loop regions of NaV channels.

## 2. Results

**Figure 1:**
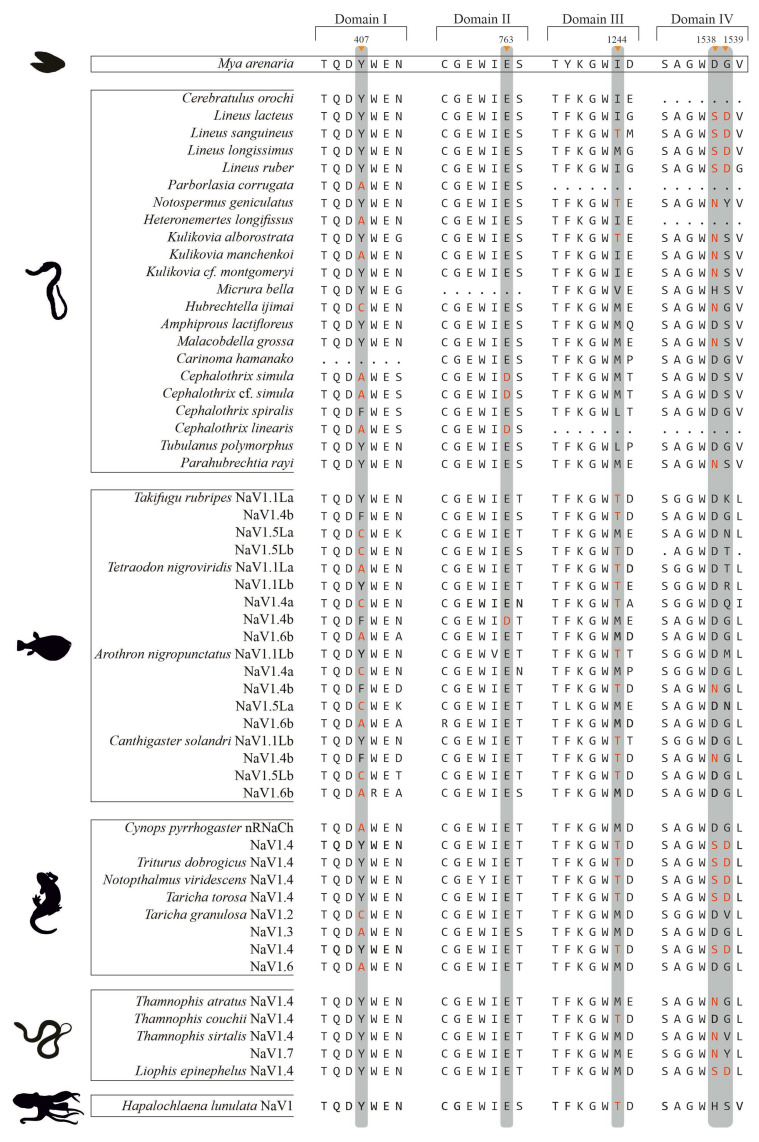
Amino acid sequences of the P-loop regions from the four domains (DI–DIV) of NaV1 channels in nonresistant *Mya arenaria* [25]*,* nemerteans analyzed in this study, and NaV isoforms from TTX-exposed species previously reported to possess substitutions affecting TTX affinity (pufferfishes [26], newts [13,27,28], snakes [12,29], and octopus [30]; references and sequence accession numbers are provided in Table S2). Positions involved in TTX binding are highlighted in gray, and residues associated with TTX resistance reported for the cited species, as well as the corresponding residues in nemerteans, which have not been experimentally validated for TTX resistance, are shown in red. A dot (.) indicates missing data.

To identify amino acid substitutions potentially conferring TTX resistance in nemertean NaV1 channels, we obtained partial amino acid sequences of the P-loop regions forming the selectivity filter from 20 species (Figure 1 and Table S1). NaV1 sequences from two additional nemertean species were included in the analysis based on previously published data [23].

In all analyzed nemertean species, the selectivity filter exhibited a typical NaV1 structure, consisting of four amino acid residues: D(406), E(760), K(1241), and A(1535). However, the amino acids forming the outer negatively charged ring of the pore (the vestibule; [24]) varied among species and often differed from the canonical EEMD motif (positions 409, 763, 1244, and 1538, respectively) (Figure 1).

## 3. Discussion

The results show that nemerteans carry substitutions at positions implicated in TTX binding across all four NaV1 domains. Because no functional studies have evaluated the TTX sensitivity of nemertean NaV channels, our interpretation of these substitutions relies on data from other TTX-resistant taxa [12,13,26,27,28,30]. Figure 1 summarizes these comparative data, incorporating non-resistant *Mya arenaria* as a reference, species in which P-loop substitutions have been experimentally tested for their effects on TTX sensitivity, and species that possess the same substitutions but have not yet been functionally assessed, including nemerteans.

It should be noted that the contribution of individual substitutions to TTX resistance has been evaluated using a range of experimental approaches, including direct electrophysiological recordings from isolated invertebrate neurons, tissues, or whole animals [12,13,28], as well as expression of mutant channels carrying the substitution of interest followed by voltage-clamp analyses [11,25,26,31,32]. Because direct functional assays of nemertean NaV channel affinity for TTX are currently lacking, our assessment necessarily relies on sequence comparisons, which can only suggest whether these substitutions exert similar effects in nemerteans.

Thus, two types of substitutions were identified at position 407 in DI: Tyr → Cys (Y407C), found in *H. ijimai*, and Tyr → Ala (Y407A), detected in six species. The Ala residue at this position has also been reported in the newt retina, where whole-cell recordings from spiking neurons demonstrated its contribution to TTX resistance [28]. Subsequent site-directed mutagenesis studies showed that replacing the aromatic residue at this site with a nonaromatic one can cause an extreme, up to 2500-fold decrease in the TTX binding affinity of NaV channels [26,32]. In DII, the Glu → Asp substitution (E763D) was observed in three nemertean species. The same substitution was previously identified in a TTX-resistant population of *Mya arenaria*, where functional assays demonstrated that it confers approximately a 3000-fold decrease in TTX sensivity in mutant channels [25,32]. It is the only substitution located in DII contributing to TTX resistance that was observed. In DIII, three species were shown to possess the Met → Thr substitution (M1244T). According to Jost and coauthors, the presence of Thr at this position increases TTX resistance by ~15-fold, as shown through site-directed mutagenesis experiments [26]. In some nemertean species studied herein, Ile, Val or Leu residues were found instead of Met, but this type of replacement is not supposed to affect TTX binding to the channel pore since all three AA sidechains provide the same characteristics. In DIV, the Asp → Asn substitution (D1538N) was identified in seven nemertean species. Functional studies have reported differing magnitudes of its effect: Geffeney and coauthors documented a ~40-fold decrease in TTX sensitivity [11], whereas Choudhary and coauthors reported an approximately 300-fold decrease [31]. Both findings were obtained using site-directed mutagenesis. Another variant in the DIV P-loop, found in four nemertean species, involves a double substitution at adjacent positions: Asp, Gly → Ser, Asp (D1538S, G1539D). This combination was previously proposed to underlie an exceptionally high (~30,000-fold) increase in TTX resistance based on intracellular recordings from isolated newt muscle fibers [13]. It should be noted that the newt species tested also carried the D1538N substitution mentioned above, which is supposed to confer much lower in TTX resistance [13].

The substitutions in key AA within the TTX-binding site that reduce TTX sensitivity have arisen independently in multiple taxa, representing a likely example of convergent evolution [12,13,26,33]. Although modifications in NaV channel structure across these lineages result from independent evolutionary events, the recurrence of the identical AA changes at the same P-loop positions suggests that there is a strictly limited set of evolutionary solutions for achieving TTX resistance without impairing channel performance [11,14]. This pattern reflects a functional limitation: only those substitutions within the TTX-binding region that do not affect normal channel operation can be allowed; the changes that lead to channel dysfunction do not persist in the population [12].

**Figure 2:**
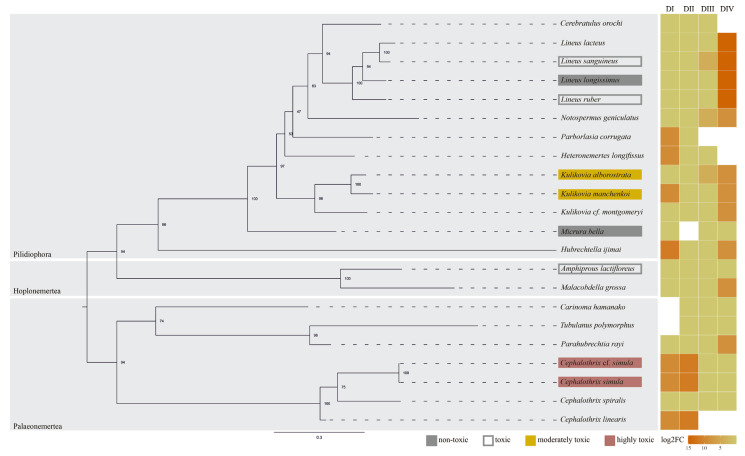
Phylogenetic distribution of amino acid substitutions in the P-loop regions of the four domains (DI–DIV) of nemertean NaV1 channels associated with TTX resistance. The tree was constructed using concatenated COI, 16S, 18S, and NaV1 gene sequences. Species are categorized as “non-toxic” (no TTX detected), “toxic” (TTX detected, concentration not determined), “moderately toxic” (TTX < 500 ng/g), and “highly toxic” (TTX > 500 ng/g). log_2_FC values represent the log_2_-transformed fold change in channel affinity to TTX for each NaV1 domain according to [11,12,13,25,26,28,31,32].

These patterns illustrate general trends of convergence across metazoans, while the distribution of substitutions within nemerteans reflects these broader trends but also reveals lineage-specific features. Nemertean substitutions presumed to affect TTX binding are spread throughout the phylum and do not form a universal pattern (Figure 2). Thus, the Y407A replacement (DI) occurs in representatives of two classes—Pilidiophora and Palaeonemertea; in DIII, the M1244T replacement appears to be characteristic of Pilidiophora; and D1538N is the only variant detected across all nemertean classes, including Hoplonemertea. At the same time, some “local clustering” could be distinguished within several nemertean genus—*Cephalothrix, Kulikovia* and *Lineus.* Three *Cephalothrix* species share the same substitutions in DI and DII, three *Kulikovia* species—in DIV, four *Lineus* species—another type of replacement in DIV. These patterns suggest that some polymorphisms could have appeared once in the common ancestor of each lineage and then distributed among its members [34].

One of the determining factors in the evolution of the TTX-binding site in tetrodotoxic animals is their exposure to TTX. Since TTX levels that can be accumulated in animal bodies vary widely, molecular adaptation of NaV channels differs as well. For example, the population of garter snake *Thamnophis sirtalis* that preys on the extremely toxic newt *Taricha granulosa* developed 10–1000 times higher TTX resistance in comparison with other populations, due to different combinations of AA substitutions in the P-loop of NaV1.4 [11,35]. TTXs levels have been quantified for only four nemertean species, and their maximum total toxins concentrations decreased in the following order: *C. simula > C.* cf. *simula > K. manchenkoi > K. alborostrata* [19,36,37]. The presumptive resistance level corresponds to their toxicity, being equal for the first two species and then decreasing in the same order.

Some limitations should be taken into account: only 8 out of 22 nemertean species were tested for TTXs [19,21,37,38,39]; moreover, the presence of TTXs is not a permanent characteristic of a species. This means that species considered TTX-free in previous studies may be able to accumulate TTXs under different conditions or in other areas. Also, the study that was shown the toxicity of several nemertean species—*L. ruber*, *L. sanguineus, A. lactifloreus* [38] utilized TTX detection methods which provide low reliability [4]. An important notion regarding the influence of P-loop structure on TTX resistance that in silico analysis of TTX binding to NaV channels of several TTX-resistant animals have demonstrated that identical substitutions appearing in distant taxa through convergent evolution do not provide equal TTX resistance, due to differences in other regions of NaV channels structures [40]. Therefore, since no investigations of nemertean NaV channels TTX resistance have been conducted, we cannot argue that all described in the current research substitutions affect the TTX binding at the same level comparing to animals in which they were originally tested. In addition, another mechanism of TTX resistance has been described in several tetrodotoxic animals, involving TTX-binding proteins that are suggested to serve as self-protection against the toxin [41,42,43]. The possible existence of similar substances in nemerteans cannot be ruled out.

## 4. Materials and Methods

### 4.1. Bioinformatic Analysis of NaV1 Sequences and Primer Design for the Selectivity Filter Regions

To identify P-loop domains of nemertean NaV1 channels in transcriptome data, we used NaV1 sequences from *Notospermus geniculatus* (accession number MZ508871) obtained in our previous study [23] and from *Lineus longissimus* (accession number XM_064765583.1) retrieved from the automatically annotated tnLinLong1.2 genome assembly (NCBI Eukaryotic Genome Annotation Pipeline; BioProject PRJEB45696) [44]. Partial sequences of the four P-loop domains of *Kulikovia alborostrata* (accession numbers MZ508867–MZ508870) were also obtained from [23].

Nav1 sequences were searched in transcriptome reads from the NCBI Sequence Read Archive (SRA) for the following nemerteans: *Lineus lacteus* (SRR3581117, SRR3581125), *Lineus sanguineus* (SRR3581110), *Lineus ruber* (SRR1324988), *Hubrechtella ijimai* (SRR1505099, SRR1505100), *Tubulanus polymorphus* (SRR1611583), *Carinoma hamanako* (SRR1505092, SRR1505094), *Cephalothrix linearis* (SRR1273790, SRR1275323, SRR1275178), *Amphiprous lactifloreus* (SRR11906528), and *Malacobdella grossa* (SRR1611560, SRR1507002). Genomic reads were also analyzed for *Cephalothrix simula* (SRR26031763) and *C. spiralis* (SRR23997129).

The quality of reads was initially assessed using FastQC v0.11.9 https://www.bioinformatics.babraham.ac.uk/projects/fastqc/ (accessed on 15 September 2022) (Babraham Bioinformatics) and cleaned of adapters and low-quality sequences using Trimmomatic v0.39 http://www.usadellab.org/cms/?page=trimmomatic (accessed on 15 September 2022) (The USAdel Lab). Cleaned reads from different runs for the same species were combined, and species-specific databases were constructed using the makeblastdb tool from the NCBI-BLAST+ v2.13.0 package https://ncbiinsights.ncbi.nlm.nih.gov/2022/03/29/blast-2-13-0/ (accessed on 30 September 2022).

Nav1-related reads were identified by aligning these databases with reference Nav1 protein sequences from *Notospermus geniculatus* (MZ508871) and *Lineus longissimus* (XM_064765583.1) using the tblastn algorithm. To exclude TTX-resistant NaV2 channels and retain only NaV1 channels, we relied on the invertebrate channel sequences reported by Boullot and colleagues [44]. Identified reads were manually assembled into contigs, which were then scanned for regions corresponding to the P-loop domains.

The P-loop regions of Nav1 from different nemertean species were aligned using MEGA 7 software https://www.megasoftware.net/ (accessed on 10 March 2025) (Table S1). Alignments were used to identify amino acid substitutions potentially affecting TTX affinity and to guide the design of primers for the amplification of Nav1 P-loop domains. Primers were initially selected using the NCBI Primer Design Tool, https://www.ncbi.nlm.nih.gov/tools/primer-blast/ (accessed on 25 May 2025)) and manually edited to produce degenerate primers where necessary.

Partial sequences from previously published work (*Kulikovia alborostrata*, MZ508867-MZ508870; *Lineus sanguineus*, *Lineus ruber*) were also included in the analysis [23].

### 4.2. Specimens Collection and Identification

**Figure 3:**
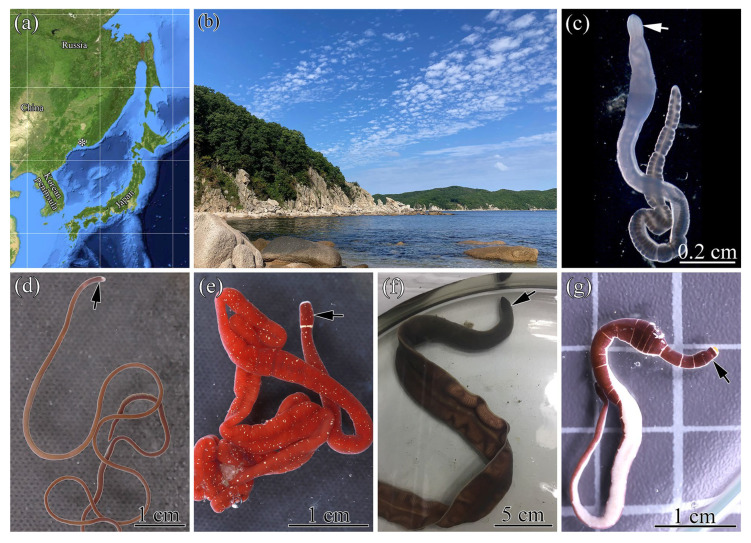
Sampling localities and live specimens of nemerteans from Spokoynaya Bay, Sea of Japan. (**a**) Geographical location of the sampling sites (asterisk). (**b**) Habitat of nemertean species. (**c**) *Parahubrechtia rayi* (photo provided by A.V. Chernyshev). (**d**) *Kulikovia alborostrata*. (**e**) *Kulikovia manchenkoi*. (**f**) *Cerebratulus orochi.* (**g**) *Micrura bella*. Arrows point to head.

**Figure 4:**
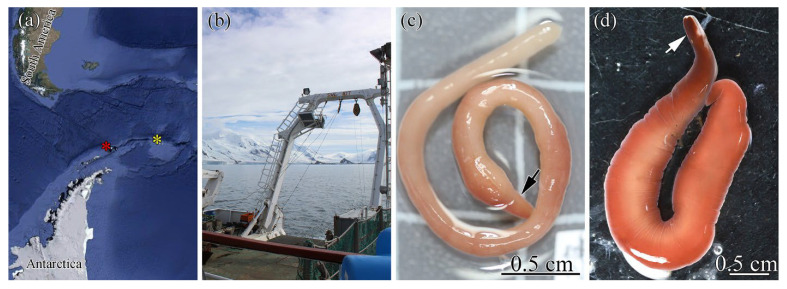
Sampling localities and live specimens of nemerteans from the Southern Ocean. (**a**) Geographical locations of the sampling sites: *Parborlasia corrugata* (red asterisk) and *Heteronemertes longifissa* (yellow asterisk). (**b**) Research vessel Akademik Mstislav Keldysh during trawling. (**c**) *Heteronemertes longifissa*. (**d**) *Parborlasia corrugata*. Arrows indicate the head.

Individuals of *Parahubrechtia rayi*, *Kulikovia alborostrata*, *K. manchenkoi*, *Cerebratulus orochi*, and *Micrura bella* were collected from the rhizoids of brown algae *Saccharina* sp. in Vostok Bay and Spokoynaya Bay (Sea of Japan) during July–August 2019–2021 (Figure 3a,b). Specimens were maintained in aquaria with seawater at 18 °C. An individual of *Kulikovia* cf*. montgomeryi* was collected in the Sea of Japan (43°22′22.8″ N, 135°20′02.4″ E) near the coast of Primorsky Krai at depths of 450–518 m, identified, and provided by A.V. Chernyshev [45]. A specimen of *Heteronemertes longifissa* was collected by A.E. Vlasenko in 2021 during the Akademik Mstislav Keldysh 79 (AMK-79) expedition to the Southern Ocean, stage 2 (Expedition ID: 6615_Sigsbee trawl) (61°29′19.0″ S, 45°53′42.6″ W), and a specimen of *Parborlasia corrugata* was collected by G.V. Malykin in 2022 during the AMK-87 expedition (Expedition ID: 7318bb-14r) (62°59′12.3″ S, 60°34′06.9″ W) (Figure 4a,b).

All specimens were collected in non-protected areas where no research access or field permits were required. Samples were fixed in RNAlater (Thermo Fisher Scientific, Waltham, MA, USA) and stored at −20 °C until further processing. Animal manipulations were performed in accordance with the ARRIVE guidelines (https://arriveguidelines.org/arrive-guidelines, accessed 14 July 2020).

Preliminary identification of all collected nemerteans was based on morphological features. For genetic identification, specimens were sequenced using the cytochrome oxidase 1 (COI) gene in all collected nemerteans, except *Cerebratulus orochi*, which was identified using 16S and 18S ribosomal RNA genes. For COI amplification, the primers LCO1490 (5′-GGTCAACAAATCATAAAGATATTGG) and HCO2198 (5′-TAAACTTCAGGGTGACCAAAAAATCA) were used [46]. For 16S amplification, the primers Ar-L (5′-CGCCTGTTTATCAAAACAT) and Br-H (5′-CCGGTCTGAACTCAGATCACGT) were used [47]. For 18S amplification, two primer sets were used: TimA (5′-AMCTGGTTGATCCTGCCAG)/1100R (5′-GATCGTCTTCGAACCTCTG) [48]; 3F (5′-GTTCGATTCCGGAGAGGGA)/9R (5′-GATCCTTCCGCAGGTTCACCTAC) [49]; and 18Sbi (5′-GAGTCTCGTTCGTTATCGGA)/18Sa2.0 (5′-ATGGTTGCAAAGCTGAAAC) [50].

PCR amplification was performed using the Encyclo Plus PCR Kit (Evrogen, Moscow, Russia), following the manufacturer’s protocol. Cycling conditions were as follows: COI: 2 min at 95 °C, followed by 38 cycles of 30 s at 95 °C, 25 s at 48 °C, 50 s at 72 °C, and a final extension of 3 min at 72 °C. 16S: 2 min at 95 °C, 38 cycles of 30 s at 95 °C, 25 s at 53 °C, 50 s at 72 °C, and a final extension of 3 min at 72 °C. 18S: 2 min at 95 °C, 40 cycles of 30 s at 95 °C, 25 s at 50 °C, 55 s at 72 °C, and a final extension of 3 min at 72 °C.

PCR products were purified using the Cleanup Standard Kit (Evrogen). Sequencing reactions were performed with forward or reverse primers and 10–20 ng of purified amplicon DNA using the BigDye Terminator v3.1 Cycle Sequencing Kit (Applied Biosystems, Waltham, MA, USA), according to the manufacturer’s instructions. Sequencing was carried out on an ABI Prism 3500 Genetic Analyzer (Applied Biosystems, Waltham, MA, USA). Resulting sequences were submitted to the GenBank database (Table 1).

### 4.3. RNA Isolation and cDNA Synthesis

Total RNA was isolated from ~15 mg of worm tissue using TRIzol reagent (Thermo Fisher Scientific, Waltham, MA, USA) following the manufacturer’s protocol. RNA concentration and purity were measured using a UV5Nano spectrophotometer (Mettler Toledo, Columbus, OH, USA). Samples with an A260/A280 ratio >1.9 were used for cDNA synthesis. Double-stranded cDNA was generated from 1 μg of total RNA using the MINT2 kit (Evrogen) and the included CDS-1 adapter, according to the manufacturer’s instructions.

### 4.4. PCR Amplification of Selective Filter Regions of the NaV1 Channel Gene

PCR amplification of the NaV1 P-loop domains was performed using primers designed in this study and from [23] (Table 2 and Table 3) with the Encyclo Plus PCR kit (Evrogen). PCR reactions consisted of 36 cycles: 94 °C for 20 s, annealing at primer-specific temperatures for 25 s, and 72 °C for 1 min. Amplicons were visualized on 2.5% agarose gels stained with ethidium bromide using GeneRuler DNA Ladder Mix (Thermo Fisher Scientific). Target bands were excised and purified using a QIAquick Gel Extraction Kit (QIAGEN, Venlo, The Netherlands), and DNA concentration and quality were assessed by UV5Nano spectrophotometry.

### 4.5. Phylogenetic Tree Construction

To construct a phylogenetic tree of the studied species, COI, 16S, 18S genes and sequences of four P-loop regions of the Nav1 were used (Table 1 and Table S1). The sequences were aligned using the L-INS-i algorithm in MAFFT version 7 [51] https://mafft.cbrc.jp/ (accessed on 23 October 2025). The analysis matrix was constructed by concatenating all three genes using SequenceMatrix [52] http://www.ggvaidya.com/taxondna/ (accessed on 23 October 2025). Phylogenetic tree reconstruction was performed using the maximum likelihood (ML) method in IQ-TREE [53] via the web server http://iqtree.cibiv.univie.ac.at/ (accessed on 23 October 2025) with 10,000 ultrafast bootstrap replicates to assess branch support [54].

## 5. Conclusions

Our study provides the first analysis of amino acid substitutions in the P-loop regions of the NaV1 channel, presumably responsible for TTX resistance in 22 species of nemerteans. Potential TTX resistance was detected in most of the studied species (16 species), including non-TTX-bearing ones, indicating that multiple evolutionary forces may be involved. Possible explanations include ecological exposure to environmental TTX or dietary interactions with tetrodotoxic prey without TTX accumulation; however, lineage-specific evolutionary changes not driven by TTX exposure cannot be excluded.

The substitutions analyzed here demonstrated no universal pattern across the phylogenetic tree, although some “local clustering” was observed within several nemertean families. These findings establish nemerteans as a valuable model for investigating the convergent molecular evolution of TTX resistance.

## Figures and Tables

**Table 1:** Nemertean species and genes with GenBank accession numbers used for phylogenetic tree construction.

Species	16S	18S	COI
*Cerebratulus orochi*	-	PX464474 *	PX472899 *
*Heteronemertes longifissus*	OQ449308	OQ449295	PX463919 *
*Hubrechtella ijimai*	KF935470	KF935303	KY986686
*Kulikovia alborostrata*	LC553790	LC553802	PX463916 *
*Kulikovia manchenkoi*	KU821490	-	PX463917 *
*Kulikovia* cf. *montgomeryi*	KU197411	-	KU197742
*Lineus lacteus*	KX261708	-	KX261759
*Lineus longissimus*	MK067321	MK076323	MK047697
*Lineus ruber*	KX261701	KY468933	GU733828
*Lineus sanguineus*	MK067331	MK076333	MK047707
*Micrura bella*	OQ449312	OQ449302	OQ450494
*Notospermus geniculatus*	LC625660	LC625685	LC625629
*Parborlasia corrugata*	EU194791	-	PX463918 *
*Carinoma hamanako*	KU197313	-	KU197661
*Cephalothrix linearis*	-	-	GU726652
*Cephalothrix simula*	OQ075733	-	PV984388
*Cephalothrix* cf. *simula*	PX559980 *	PX586769 *	PX526497 *
*Cephalothrix spiralis*	KU197338	-	GU726648
*Parahubrechtia rayi*	-	-	PX463920 *
*Tubulanus polymorphus*	JF277598	JF293061	KU197697
*Amphiprous lactifloreus*	MN211511	MN211417	MN205528
*Malacobdella grossa*	MZ231135	MZ231197	MZ216519

*: Current paper, -: no available data.

**Table 2:** List of primers used in this study. Forward primer sequences are shown in bold.

	**№**	**Primer Name**	**Primer Sequence**	**Reference**
DI	1	DIFUni	**RTGCGCMTTYCGMCTYATGAC**	Current study
	2	DI_Forward	**ATGCGCCTTTCGCCTTATGAC**	[23]
	3	DI_Reverse	CGGCGTTCTTCCTCTTCCTTT	[23]
	4	RID_improved	CATTCTGATGGACTTTTTGGCA	Current study
	5	DIFw_csim	**GTTTTTCAGTCAATGTTTGG**	Current study
	6	DIRev_csim	CGACACTTACTAACTGATAC	Current study
	7	DIRUni	CGGCGYTCYTCYTCTTCCTTT	Current study
DII	8	DIIForward	**GTCCTYCGAACATTCAGATTGC**	[23]
	9	DII reverse	AGATTGGAGATTTTCAGCCCC	[23]
	10	DII reverse v2	ATGCTTTCAATCCATTCCCCA	Current study
	11	DIIFw_csim	**TTCATATTTGCTGTCGTCGGT**	Current study
	12	DIIRev_csim	CTAACACCAAGTCCCCTCAAC	Current study
DIII	13	DIII forward	**GTCTTCTGGCTCATCTTCAGTATCA**	[23]
	14	DIII reverse	TCAGCGTGAAGAAAGAACCGA	[23]
	15	DIIIFw_paleo	**CTGGCTKATCTTTAGYATMATGGG**	Current study
	16	DIIIRev_paleo	TACCCCTCTCATRTCSGTYG	Current study
DIV	17	DIV Forward	**AACATGCTGCCGGGATAGA**	[23]
	18	DIV Forward new	**CGCTAGCGGTTTCACTTCCT**	Current study
	19	DIV reverse	TTGCCGCAGTTACCCTTGAC	[23]
	20	DIV_F_paleo	**TCCCTGCCTGCCYTMTTC**	Current study
	21	DIV_R_paleo	GTACTGCGTGGCTTCAGGATC	Current study

**Table 3:** Primer combinations used for amplification of the four P-loop regions of the nemertean NaV1 channel.

	**DI—Primer Combinations**	**DII—Primer Combinations**	**DIII—Primer Combinations**	**DIV—Primer Combinations**
*Cerebratulus orochi*	DIFUni + RID_improved (Ta 57 °C) len 281bp	DIIForward + DII reverse (Ta 57 °C) len 431bp	DIII forward + DIII reverse (Ta 57 °C) len 348bp	N/A
*Heteronemertes longifissus*	DI_Forward + DI_Reverse (Ta 56 °C) len 233bp	DIIForward + DII reverse (Ta 57 °C) len 431bp	DIII forward + DIII reverse (Ta 57 °C) len 348bp	N/A
*Kulikovia manchenkoi*	DI_Forward + DI_Reverse (Ta 56 °C) len 233bp	DIIForward + DII reverse (Ta 57 °C) len 431bp	DIII forward + DIII reverse (Ta 57 °C) len 348bp	DIV Forward + DIV reverse (Ta 56 °C) len 193bp
*Kulikovia* cf. *montgomeryi*	DI_Forward+ DI_Reverse (Ta 56 °C) len 233bp	DIIForward + DII reverse (Ta 57 °C) len 431bp p	DIII forward + DIII reverse (Ta 57 °C) len 348bp	DIV Forward + DIV reverse (Ta 56 °C) len 193bp
*Micrura bella*	DI_Forward+ DI_Reverse (Ta 56 °C) len 233bp	N/A	DIII forward + DIII reverse (Ta 57 °C) len 348bp	DIV Forward new + DIV reverse (Ta 57 °C) len 294bp
*Parborlasia corrugata*	DIFUni +DIRUni (Ta 56 °C) len 233bp	DIIForward + DII reverse v2 (Ta 56 °C) len 295bp	N/A	N/A
*Parahubrechtia rayi*	DIFUni+ DI_Reverse (Ta 52 °C) len 233bp	DIIForward + DII reverse v2 (Ta 56 °C) len 295bp	DIII forward + DIII reverse (Ta 57 °C) len 348bp	DIV Forward + DIV reverse (Ta 56 °C) len 193bp
*Cephalothrix* cf. *simula*	DIFw_csim + DIRev_csim (Ta 47 °C) len 163bp	DIIFw_csim + DIIRev_csim (Ta 52 °C) len 334bp	DIIIFw_paleo + DIIIRev_paleo (Ta 57 °C) len 270 bp	DIV_F_paleo DIV_R_paleo (Ta 53 °C) len 464bp

## Data Availability

The original contributions presented in this study are included in the article/Supplementary Materials. Further inquiries can be directed to the corresponding author.

## Supplementary Materials

The following supporting information can be downloaded at: https://www.mdpi.com/article/10.3390/ijms262411785/s1.
