# Supplementary material for: Voltage-Gated Sodium Channel Substitutions Underlying Tetrodotoxin Resistance in Nemerteans: Ecological and Evolutionary Implications
Source: Int J Mol Sci. 2025 Dec 5;26(24):11785. doi: 10.3390/ijms262411785 (PMC12732450; doi:10.3390/ijms262411785)
Supplement: Supplementary file 1 [file ijms-26-11785-s001.zip › Table_S2.pdf]

Species	NaV channel	Reference	Uniprot accession number	Genbank accession number
Takifugu rubripes	NaV1.1La	[1]		XP_029698008.1
	NaV1.1Lb	[1]		
	NaV1.4b	[1]	Q2XVR6	ABB29442.1
	NaV1.5La	[1]		XP_029687042.1
	NaV1.5Lb	[1]		XP_029698632.1
Tetraodon nigroviridis	NaV1.1La DI, DIII, DIV	[1]	H3BY44	
	NaV1.1La DII	[1]		
	NaV1.1Lb	[1]	H3C1K3	
	NaV1.4a	[1]	Q2XVR5	DQ221251.1
	NaV1.4b	[1]	Q2XVR4	DQ221252.1
	NaV1.6b	[1]		
Arothron nigropunctatus	NaV1.1Lb	[1]		EU391401.1
	NaV1.4a	[1]		EU391403.1
	NaV1.4b	[1]		EU391404.1
	NaV1.5La	[1]		EU391405.1
	NaV1.6b	[1]		EU391402.1
Canthigaster solandri	NaV1.1Lb	[1]		EU391407.1
	NaV1.4b	[1]		EU391408.1
	NaV1.5Lb	[1]		EU391409.1
	NaV1.6b	[1]		EU391411.1
Cynops pyrrhogaster	nRNaCh	[2]	Q9YGN7	AF123593.1
	NaV1.4	[3]	A0A0A0Y2W6	KP118971.1
Triturus dobrogicus	NaV1.4	[3]	A0A0A0Y194	KP118973.1
Notopthalmus viridescens	NaV1.4	[3]	A0A0A0Y2B8	KP118970.1
Taricha torosa	NaV1.4	[3]	A0A0A0Y190	KP118968.1
Taricha granulosa	NaV1.2	[4]	A0A6G9W1G2	MT125669.1
	NaV1.3	[4]	A0A6G9W273	MT125670.1
	NaV1.4	[4]	A0A0A0Y7I0	KP118969.1
	NaV1.6	[4]	A0A6G9W1G2	MT125669.1
Thamnophis atratus	NaV1.4		C7AK99	FJ570810.1
	NaV1.4 DIII, DIV	[5]	C7AK99	FJ570810.1
Thamnophis couchii	NaV1.4		C7AKA1	FJ570812.1
	NaV1.4 DIII, DIV	[5]	C7AKA1	FJ570812.1
Thamnophis sirtalis	NaV1.4	[6]	A0A1W5T0H6	KY745667.1
	NaV1.7	[6]	A0A6I9X9N4	NW_013658040.1
Liophis epinephelus	NaV1.4 DI		H9E9G4	JQ687547.1
	NaV1.4 DII		H9E9P9	JQ687632.1
	NaV1.4 DIII	[5]	H9E9Y2	JQ687715.1
	NaV1.4 DIV	[5]	H9EA57	JQ687790.1
Hapalochlaena lunulata	NaV1	[7]	A0A7S9VLQ3	QPI69428.1

References

1. Jost, M.C.; Hillis, D.M.; Lu, Y.; Kyle, J.W.; Fozzard, H.A.; Zakon, H.H. Toxin-resistant sodium channels: Parallel adaptive evolution across a complete gene family. *Mol. Biol. Evol.* **2008**, *25*, 1016–1024, doi:10.1093/molbev/msn025.

2. Kaneko, Y.; Matsumoto, G.; Hanyu, Y. TTX resistivity of Na+ channel in newt retinal neuron. *Biochem. Biophys. Res. Commun.* **1997**, *240*, 651–656, doi:10.1006/bbrc.1997.7696.

3. Hanifin, C.T.; Gilly, W.F. Evolutionary history of a complex adaptation: Tetrodotoxin resistance in salamanders. *Evolution (N. Y).* **2015**, *69*, 232–244, doi:10.1111/evo.12552.Evolutionary.

4. Vaelli, P.M.; Theis, K.R.; Williams, J.E.; O'connell, L.A.; Foster, J.A.; Eisthen, H.L. The skin microbiome facilitates adaptive tetrodotoxin production in poisonous newts. *Elife* **2020**, *9*, 1–29, doi:10.7554/eLife.53898.

5. Feldman, C.R.; Brodie, E.D.; Brodie, E.D.; Pfrender, M.E. Constraint shapes convergence in tetrodotoxin-resistant sodium channels of snakes. *Proc. Natl. Acad. Sci. U. S. A.* **2012**, *109*, 4556–4561, doi:10.1073/pnas.1113468109.

6. McGlothlin, J.W.; Chuckalovcak, J.P.; Janes, D.E.; Edwards, S. V.; Feldman, C.R.; Brodie Jr, E.D.; Pfrender, M.E.; Brodie III, E.D. Parallel evolution of tetrodotoxin resistance in three voltage-gated sodium channel genes in the garter snake Hamnophis sirtalis. *Mol. Biol. Evol.* **2014**, *31*, 2836–2846, doi:10.1093/molbev/msu237.

7. Geffeney, S.L.; Williams, B.L.; Rosenthal, J.J.C.; Birk, M.A.; Felkins, J.; Wisell, C.M.; Curry, E.R.; Hanifin, C.T. Convergent and parallel evolution in a voltage-gated sodium channel underlies TTX-resistance in the greater blue-ringed octopus: *Hapalochlaena lunulata*. *Toxicon* **2019**, *170*, 77–84, doi:10.1016/j.toxicon.2019.09.013.
